# Start-up financing of professional pest control in pig farming in North Rhine-Westphalia in Germany

**DOI:** 10.1186/s40813-018-0099-0

**Published:** 2018-10-01

**Authors:** Odile C. Hecker, Marc Boelhauve, Marcus Mergenthaler

**Affiliations:** Department of Agriculture, South Westphalia University of Applied Sciences, Lübecker Ring 2, 59494 Soest, Germany

**Keywords:** Pest control, Start-up financing, Pest control operator, Pig farming, Anticoagulant rodenticide, Implementation practice, Environmental risk

## Abstract

**Background:**

Due to the risk of spreading epizootic diseases through rodents, pest control is mandatory in pig farming in European countries. However, there is limited research focused on rodent control practices, usage of anticoagulant rodenticides, and the acceptance of Pest Control Operators (PCOs) in pig farming in Germany. Therefore, the present study aims to investigate current control practices in pig holdings and to analyze the potential of a financial support on the implementation of professional pest control.

**Results:**

Data were collected from monitoring records of PCOs and personal interviews with farmers and PCOs. 33 of 47 farmers, who were offered the possibility to outsource rodent control to PCOs supported by financial contribution of the North Rhine-Westphalian Animal Disease Fund (TSK) for a period of 2 years, joined the project.

Despite the widespread opinion that the professional would not be beneficial – the authors figured out that farmers could financially benefit in time saved and by improved rodent control measures from the work of the PCOs. Costs of pest control measures per operation on average did not differ significantly between costs incurred by employment of PCOs (1.310 € per year) and calculated costs that arise by farmers themselves (1.217 € per year).

All PCOs used Difenacoum and Brodifacoum against pest infestations. In doing so, the infestation with rodents was reduced and most of the participating farmers assessed the project as successful and employ the PCOs permanently. However, mapping the farm locations to resistance areas of the Rodenticide Resistance Action Committee (RRAC) shows that Brodifacoum was frequently used in areas which are marked as areas that are at low risk or rather have no risk for resistance. The environmental risks, however, are increased in these areas.

**Conclusion:**

The instrument of temporal start-up financing professional pest control allows ensuring the continuous engagement of PCOs after the project period. This could possibly lead to long-term effects on the individual farm hygiene and on disease prevention. Nevertheless, important research questions with regard to the application of anticoagulant rodenticides of farmers and PCOs in livestock farming and with regard to risk mitigation measures were generated, meriting further investigation.

**Electronic supplementary material:**

The online version of this article (10.1186/s40813-018-0099-0) contains supplementary material, which is available to authorized users.

## Background

Rodent pests cause numerous damages to agricultural operations. Most importantly, rodents are carriers of various pathogens that cause human and animal diseases, such as leptospirosis, toxoplasmosis, q-fever, and gastroenteritis caused by salmonella and campylobacter spp. [1–5]. Taken into account that populations of up to 15.000 rats have been counted in large pig production units in Germany and that rats might roam up to 1.5 km within a few hours thereby being able to visit different livestock farms in livestock dense regions, the risk of spreading epizootic diseases must be considered as high [6, 7]. Furthermore, rodents cause considerable economic damages including increases in feed costs through the consumption and contamination of feed as well as damage to electrical equipment and other facilities [8]. Most of all, the steady availability of feed, water, retreats as well as high stocking densities and deficits in hygiene make livestock production systems particularly attractive to rodents [9]. Therefore, the control for neophobic rodent populations by anticoagulant rodenticides is common and widespread. The development of a resistance against some first generation anticoagulant rodenticides (FGARs) in rats and mice about 60 years ago led to the introduction of a group of anticoagulant, which have increased potency, prolonged biological half-lives, and hepatic accumulation abilities [10–12]. The second generation anticoagulant rodenticides (SGARs), namely brodifacoum, difenacoum, flocoumafen, bromadiolon, and difethialon can be a highly effective means of controlling rodent populations and therefore were used in great frequency against rats and mice in the past [13–15]. Advantages of SGARs are the delayed action of these compounds with mortality appearing 3 to 7 days after bait consumption, which makes them effective in pest controlling [16]. Nonetheless, resistance to the SGARs Bromadiolon and Difenacoum was described in many countries worldwide such as Germany. Here the resistance occurred in brown rats (*R. norvegicus*) for large-scale areas in Northwest Germany as well as in mice (*Mus musculus*) for many locations throughout Germany [17, 18]. In addition, due to their increased tissue persistency, the unintended exposure of non-target mammals and birds, either directly via consumption of bait (primary exposure) or indirectly by consumption of an animal that has already been exposed (secondary exposure), is commonly described in wildlife [14, 19, 20]. The high risk of non-target poisoning by SGARs is taken into account in the Biocides Directive (EU) No. 528/2012. FGARs may still be used by consumers, but their use is restricted to the private, domestic sphere, i.e. indoor areas and outdoor areas immediately around buildings. In contrast, the use of SGARs and that of FGARS in certain scenarios (i.e. open areas) is limited to persons with a certificate of competence. This includes the qualification under Annex I No. 3.4 German Ordinance on Hazardous Substances (GefStoffV) for PCOs as well as qualifications under the German Ordinance Specialist Qualification in Plant Protection (PflSchSachkV) for farmers. Therefore, farmers with qualifications also are professional users [21]. The need for rodent control measures to prevent damages on agricultural operations on the one side, and the recognized risk of development of resistances and secondary poisoning for predators of rodents on the other side, makes the usage of anticoagulant rodenticide (AR) a double-edged sword. In livestock farming, efficient pest control often requires placing baits (applied in bait stations) both in and around buildings as well as in outdoor areas, which are limited to professional users with qualification and to PCOs. Though most of the farmers are formally authorized to use SGAR. Implementations of effective rodent control schemes remain challenging for non-PCOs as control measures have to be performed steadily even in times of workload peaks on farms. Unfortunately, studies about the implementation practices of rodenticide pest control measures in pig farming in Germany are missing. The objective of the current study is to provide farmers an easy entry to outsource rodent control to PCOs by a start-up financial contribution of the North Rhine-Westphalian Animal Disease Fund (TSK). Therefore, rodent control was implemented by PCOs. To improve the effective sustainability planning, customer satisfaction as well as criticism were recorded for both farmers and PCOs. By scientifically monitoring this project, we aimed to identify and optimize, when required, current usage practices on pig farming with the goal of increasing the effectiveness of rodent control by the implementation of best user practices by PCOs.

## Methods

### Pig farms

This study was conducted on pig farms in North Rhine-Westphalia in Germany from August 2014 to October 2017. In total, 47 farmers were offered the possibility for the duration of 2 years to outsource rodent control to pest control operators (PCOs) supported by a start-up financial contribution of the North Rhine-Westphalian Animal Disease Fund (TSK). The current project is part of the project “Implementation of measures to improve animal hygiene and to prevent epizootic diseases in pig farming in North Rhine-Westphalia in Germany”. Within this project, the South Westphalia University made a call in October 2013 to farmers in the Region of North Rhine-Westphalia to join one of two workshops via regional advisors of different organizations and veterinarians. Potential participants for the overall project were identified during these two kick-off workshops, which were used to inform the farmers about the project contents. Farmers within the project were informed in July 2014 by letters about the partial project on pest control and could chose based on their willingness to participate in the study. The amount of the subsidy was 1.000 € for each farm per year. Total cash costs of hiring PCOs per farm differed depending on the individual farm conditions between 1.054 € and 2.920 €. Cash costs that exceed the financial subsidies (x̅ = 395 € ± 354 €) were paid by the farmers themselves. Data on farm structures and socio-demographic details on farmers were available from former data collection within the project.

Phone interviews with the farmers were conducted in the first phase of the project in July 2014 to assess the factors that affect the contribution (or not) to the pest control program as well as key considerations in the decision which company of pest control operation was to be chosen. The interviews were based on a standardized questionnaire. After the planned stopping of the financial contribution, personal on-site interviews were conducted with the contributing farmers from December 2016 until February 2017. The questionnaire consisted of open and closed questions. The questions related to project success, perception of the joint activities, the financial evaluation of pest control measures, and further employment of PCOs. In addition, 10 to 14 months after the end of the financial contribution, the long-term collaboration of farmers with the PCOs was evaluated in a final phone interview with the farmers (10/2017) based on a standardized questionnaire.

To classify the farm structure, the stage of production was described as follows: Sow breeding farms are defined as units that keep sows, irrespective of whether there is also piglet breeding and/or pig fattening or none of the before mentioned. Piglet rearing farms are determined as operations without sow keeping but irrespective of whether there is fattening or not. Fattening holdings are defined as units keeping only fattening pigs without sow breeding and/or piglet rearing. For a comparison of the farms, existing data of animal numbers were converted into livestock units and summed up. Resistance risk levels for rats and mice against anticoagulant rodenticides at farm sites were assessed through the location of the farms using the web-site of the Rodenticide Resistance Action Committee (RRAC). Socio-demographic details on farmers included factors such as age, gender, and vocational education. Thereby, it was recorded whether the farmer has a training qualification inside or outside the agricultural sector. In addition, the highest vocational training was assessed as an agricultural training qualification. In doing so, three categories of qualifications were distinguished: (1) basic education as farmer, (2) state certified farmer or technician, and (3) degree of a university or university of applied sciences. Farmers self-selected one of the two groups: one group of farmers which hired a PCO and the other that did not. The group of farmers working together with a PCO was further divided into farmers who employed the PCO for the time of the project and those who went further by working together with the PCO even beyond the end of the financial support.

### Pest control program

The intervention was carried out on participating farms and was conducted for 2 years. All PCOs working on the project were trained as professional users having a specialist qualification, i.e., in the form of an officially recognized training or advanced training in the respective field. PCOs were chosen by the farmers out of an established list of companies according to these predefined quality criteria.

The frequency of visiting individual farms was pre-specified within the project. During the first 3 months of the project, PCOs visited the farms as often as necessary but at least every 2 to 3 weeks. Thereafter, the visiting frequency was adjusted within a range of four to 6 weeks to the individual needs of each farm. The active substance, the amount, and the formulation of the baits as well as the number of baiting points were chosen and documented by PCOs. Recorded data of PCOs included all documents of the rodent control operations (i.e., site plan, documentation of each visit). The documented quantities of the baits used were included in the study as well as if the control period of the farm was 10 months or longer and if the quantity of bait used was documented for, at least, every second visit. Information provided by PCOs for 21 farms met these criteria and were analyzed.

In addition, personal on-site interviews were conducted with PCOs in February 2016 to assess the situation at the beginning of the project, the course of the project as well as the perception of joint activities. Performance indicators of pest control measures at beginning of the project were considered. Thereby, the extent of the infestation with rodents was surveyed from PCOs viewpoint on the 5 point Likert scales based on three questions (extent of problems with rodents, extent of infestation with mice and with rats). Mean values of manifestations were re-scaled to values between zero and four so that high values stand for high infestations with rodents.

Furthermore, preventive measures were part of the pest control program. PCOs were required to draw attention to three dimensions: Firstly, building defects, secondly, waste and garbage, and thirdly, uncontrolled plant growth on operations. Implementation practices of preventive measures were assessed by PCOs in personal on-site-interviews in February 2016. The question “Were the terms of your agreement you made with the farmer kept?” was surveyed based on the three dimensions mentioned above by the 5 point Likert scales. The data was re-coded from zero to four so that high values stand for a high implementation practice. The data was analyzed using descriptive statistics and calculation of Pearson correlation coefficients.

### Data analysis

To analyze metric variables, calculated mean values were tested for statistical significance between groups by ANOVA. To test for independence of rows and columns in contingency tables the Fisher exact test was conducted.

Categorical variables were compared using Mann-Whitney U test for non-normally distributed data. For statistical comparison of time durations, a one-tailed Kruskal-Wallis rank sum test was used. For statistical analysis, the software SPSS Statistics 21 (IBM Deutschland GmbH, Ehningen, Germany) was used. The significance level for all statistical tests was set at α = 0.05.

## Results

### Project participation and employment of PCOs

Of 47 farmers who were offered to participate, 33 (70%) employed a PCO. Seven farmers already employed a PCO for a longer time yet with undefined interventions irrespective of the current project. Interview information was provided by 32 of 33 farmers who employed a PCO and was analyzed (as one farmer was not available for an interview). Of the 14 (30%) non-participating farms, eleven farmers completed a survey with a reduced number of questions related to pest control.

Farms were distributed in the North and East of North Rhine-Westphalia (Additional file 1: Figure S1). They included several types [sow-keeping (*n* = 2), piglet-breeding (*n* = 3), fattening farms (*n* = 24) as well as a combination of these: sow keeping and piglet rearing (*n* = 7), piglet rearing and fattening (*n* = 4), closed system (*n* = 5)]. Sizes varied from 72 to 1040 ($\overline{x}$= 301) livestock units (Table 1). Based on the classification defined in the Methods Section, the study population was composed of 14 sow breeding farms, 7 piglet breeding farms, and 24 fattening farms. Within the category “sow breeding”, 86% of the farmers employed a PCO during the project, whereas 43% of the farmers in the category “piglet breeding” and 75% in the category “fattening farms” did so. Besides pig farming, there was other animal farming (poultry, cattle, or horses) on five farms.

**Table 1:** Farm structure of holdings and level of participation

Farm structure	Number	LSU, x̅	LSU (Min.- Max.)	Employment of PCO (%)
Category sow breeding	14	218	120–346	86
Sow breeding	2	150	150–150	0
Sows and piglets	7	228	144–336	100
Closed system	5	232	120–346	100
Category piglet breeding	7	229	72–478	43
Piglet rearing	3	120	72–168	0
Piglets and fattening	4	311	228–478	75
Category fattening farms	24	370	96–1040	75
Total	45	301	72–1040	73

Farmers, who engaged a PCO for a period longer than the two-year program, answered, when asked for their motives, that the infestation with rodents was the main point to do so (86%). Other incentive reasons were the realization of the importance of pest control measures (57%) and the recognition of the proficiency of the PCO (57%). Time advantages (29%), the comparison of benefits and costs, and the openness for novelty (14% each) were less commonly cited. In contrast, farmers who decided to hire a PCO as part of the project stated the partial financing was the primary reason to do so (64%). Realizing the importance of pest control measures was mentioned by half of the farmers as well as the requests of South Westphalia University (46%). Current infestation with rodents, openness for novelty, the guaranteed quality standards, and the nomination of specific providers were less frequently named.

Farmers who refused the employment of a PCO despite the services offered, mentioned the low infestation with rodents as the main reason (70%). Other reasons for refusal named by half of the farmers were the high estimation of their own skills, whereas the comparison of benefits and costs played a minor role for the decision.

**Fig. 1:**
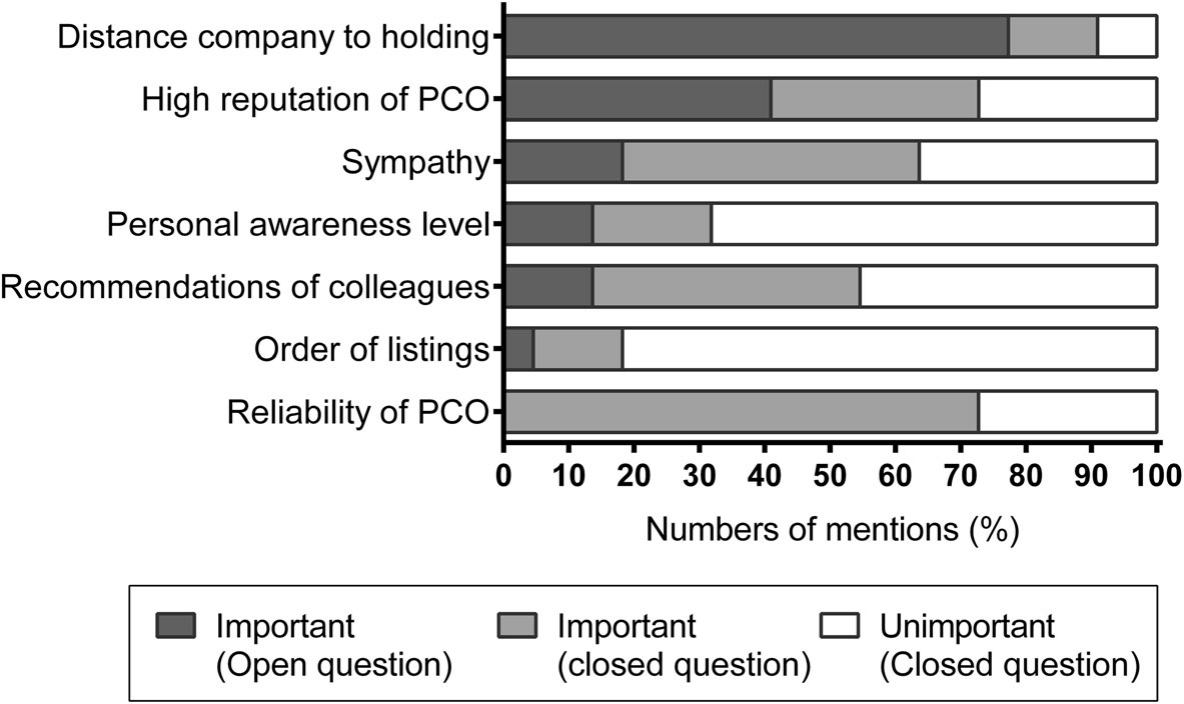
Numbers of mentions of recruitment criteria of farmers for pest control operator companies (*n* = 22)

During the engagement of PCOs, the operation managers could choose out of an established list of five companies. When asked in an open question for the key considerations in the decision which company to choose, almost 70% farmers answered that the distance of the company to the holding was essential for their choice (Fig. 1). The combination of open and choice-based questions revealed that half of the farmers mentioned the high reputation of the PCOs as well as the sympathy and the recommendation of colleagues as major reasons. Furthermore, reliability was mentioned by approximately three-fourths of farmers as an important employment criterion. In contrast, the personal awareness level of the PCO and the order of listings were more or less irrelevant for choosing a PCO company by most of the farmers (Fig. 1).

### Pest control measures on pig farms

**Fig. 2:**
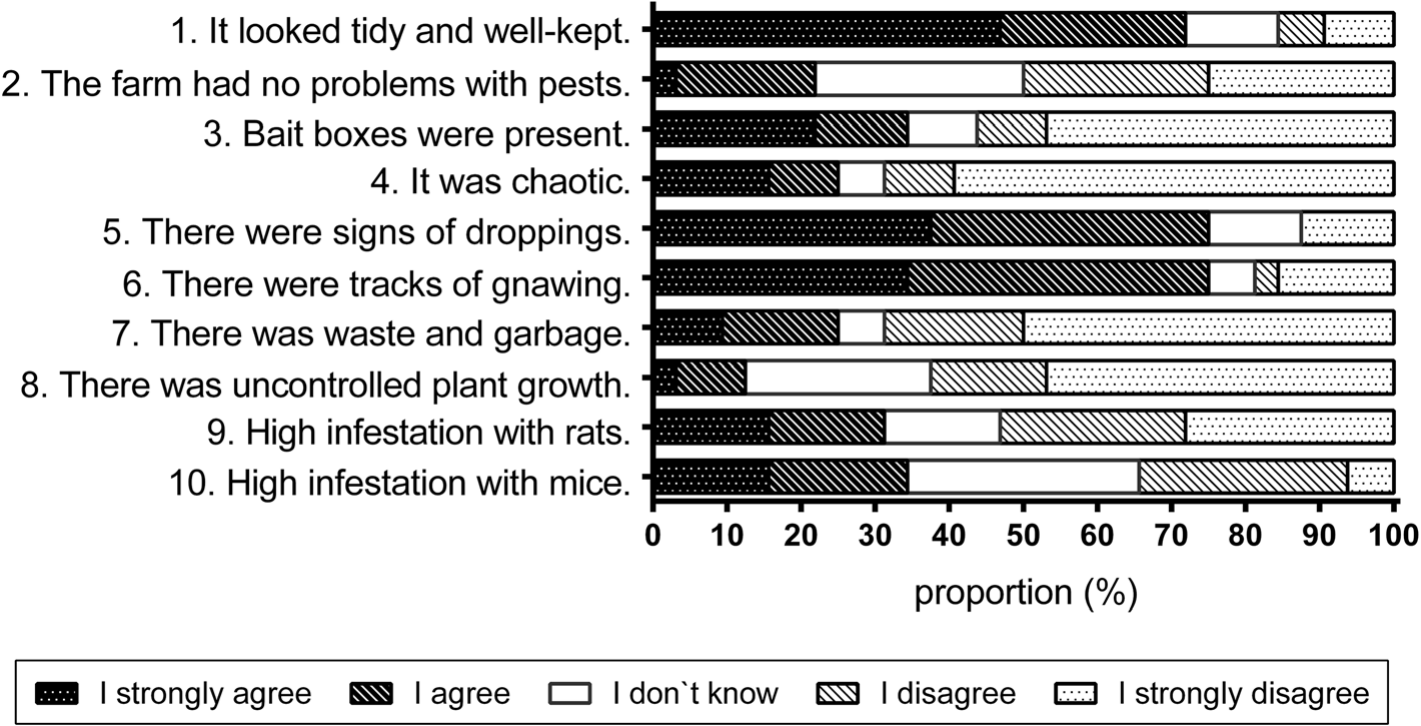
Assessment of the situation before the project was started by PCOs in regard to pest control measures in selected animal holdings (*n* = 32)

PCOs assessed the situation before the project was started regarding pest control measures in 32 animal holdings within personal on-site interviews in February 2016 (Fig. 2). Basically, 23 (72%) holdings were described as tidy and well-kept. From the PCOs perspective one fifth of the operations had no problems with rodent pests. High numbers of infestation with mice and rats were described on eleven (34%) and ten (31%) farms, respectively. In general, the infestation with rats and mice was estimated by PCOs ranging from low to medium high prevalence rates (Table 2). On sow breeding farms, PCOs assessed the infestation with rats as being highest, whereas it was assessed lowest on piglet breeding farms (Table 2). The infestation with mice was estimated as being higher on sow breeding and fattening farms as compared to piglet breeding farms. Overall, PCOs assessed the extent of problems with rodents as medium size, with reduced problems on piglet breeding farms in comparison to sow breeding and fattening farms (Table 2). There were no statistically significant differences in pest infestation between the different farm categories. In 18 holdings (56%), no bait boxes were present at all. Tracks of droppings and gnawing were present on 24 farms (75%), whereas waste and garbage lying around was present on 25% of the farms. Excessive plant growth close to the stables has been described at 13% of the holdings. Farms within this study are all located in North-West-Germany. We checked for resistance risk levels at farm sites. For seven farms there was no risk of resistance and sixteen units were located in areas of low risk. In addition seven units were at medium risk and 4 farms at high resistance risk level due to their facility location. Assessing the situation of infestation by means of the recorded data of PCOs, 8 % of the holdings were infested merely with mice, whereas 86% of the holdings were infested by both mice and rats. In 6 % of the operations the information on the infestation was missing. Against these infestations all PCOs used anticoagulant rodenticides on all farms. Most commonly SGARs were used (Table 1) and accounted for 94% of applications (406 of 432). The quantity data is expressed in terms of the product which was mainly food bait as well as active ingredient. The most commonly used active ingredient of SGARs was Brodifacoum followed by Difenacoum. These two rodenticides cumulatively accounted for 98% of amounts of active ingredients of SGARs used within this study. Brodifacoum was the most common used anticoagulant in all seven farms located in areas which are at no risk for resistance. Likewise, on farms that were at low resistance risk level, Brodifacoum was used as the main compound on 14 of 16 pig farms. On farms that were at a medium and high resistance risk level, pest control measures were carried out using Brodifacoum most frequently except in one case where Difenacoum was most commonly used.

**Table 2:** Average values of infestation with rodents of different farm categories assessed by PCOs at the beginning of the project

Farm structure	Number	Infestation with rats x̅ ± SD	Infestation with mice x̅ ± SD	Extent of problems with rodents x̅ ± SD
Category sow breeding	11	2.1 ± 1.5	1.9 ± 1.2	2.1 ± 1.2
Category piglet breeding	3	0.3 ± 0.6	1.0 ± 1.5	1.1 ± 0.2
Category fattening farms	18	1.6 ± 1.4	2.1 ± 1.2	2.2 ± 1.0
Total	32	1.7 ± 1.5	2.0 ± 1.2	2.0 ± 1.1

Values range from zero to four; high values stand for high infestation with rodents

Flocoumafen and a combination product consisting of Bromadiolon and Difenacoum were used to a lesser extent (Table 3). The only FGAR used was Coumatetralyl in the form of foamed material. During the analysis period, grain-based baits were the most commonly used formulation in pig farming (68% of use by weight), followed by paste baits (26% of use by weight) and baits in the form of foamed material (6% of use by weight). Besides the rodent control with chemical rodenticides, snap trapping was additionally carried out on two farms.

**Table 3:** Average quantities of rodenticide products and active ingredients applied monthly per pig farming unit in North Rhine-Westphalia in Germany of the period 2014–2016 (*n* = 21 pig farming units; *n* = 378 protocols)

FGARs/SGARs	ø used quantities of rodenticide products applied per farm per month	ø used quantities of active ingredients applied per farm per month
Coumatetralyl	22.1 ± 69.3 ml	88.5 ± 277.3 mg
Warfarin	–	–
Chloro- and Diphacinon	–	–
Brodifacoum	813.2 ± 930.2 g	50.1 ± 62.8 mg
Difenacoum	143.4 ± 218.6 g	7.2 ± 10.9 mg
Flocoumafen	13.3 ± 50.1 g	0.7 ± 2.5 mg
Bromadiolon + Difenacoum	3.2 ± 9.6 g	0.1 ± 0.2 mg (each)
Difethialon	–	–

In addition to chemical control of pests by rodenticides, preventive measures were part of the pest control program. PCOs were required to draw attention to mainly three dimensions (building defects, waste and garbage as well as uncontrolled plant growth). Agreements in terms of the removal of waste and garbage were kept in 87% and in terms of the removal of building defects in 61% of the farms (Table 4). Less frequent (in 57% of the farms) agreements in terms of the removal of uncontrolled plant growth were kept. The average manifestation of implementation practices is about 2.5 from a range of possible values between zero and four. The strongest implementation practice of measures was in terms of the removal of uncontrolled plant growth whereas it was lowest in terms of the removal of building defects (Table 4).

**Table 4:** Average manifestation of implementation practices in terms of removal of building defects, waste and garbage, and uncontrolled plant growth (4 = high implementation, 0 = low implementation)

Removal of...	N	%	μ	σ
...building defects	14	61	2.29	1.64
...waste and garbage	20	87	2.60	1.70
...plant growth	13	57	2.77	1.48
Total	23	100	2.48	1.41

**Fig. 3:**
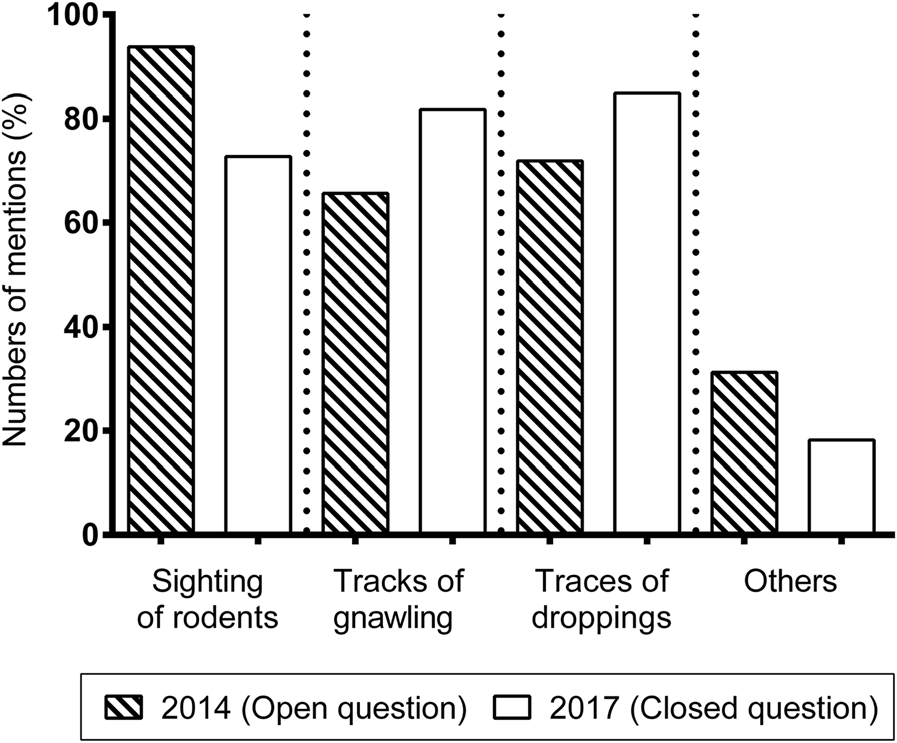
Comparative presentation of numbers of mentions of pest infestations indicators by farmers of pig farming units in percent before the project started (2014; *n* = 32) and after 2 years of collaboration with PCOs (2017; *n* = 32)

As an indicator of the success of the project, the awareness of farmers regarding relevant indicators of rodent populations was assessed during the project. In an open question, almost 95% of the farmers interviewed in 2014 mention that the sighting of rodents is an important indicator to act against rodents (Fig. 3). Traces, such as remnants of feeding and droppings (66 and 72%) as well as other signs, e.g., run marks (31%) were mentioned considerably less frequently. Three years later farmers were asked again (in a closed question) for signs of rodents. This time the sighting of rodents was mentioned as noticeably less frequent as an indicator to act, whereas the proportion of farmers who took traces in the form of remnants of feeding and droppings into account has considerably increased to 82 and 85% (Fig. 3). In 2014 farmers stated specific diseased animals as evidence of a manifestation, for instance infections with salmonella sp. By Contrast, in 2017 run marks of animals on different surfaces were named.

### Long-term participation of farmers and costs of pest control measures

Most farmers agreed that the project was successful when answering the question of whether the project was successful or not from the point of view of participating farmers. More than half of the farmers thought the success of the project was due to the reduction of the infestation of rats and mice along with reduced tracks of gnawing. Further positive aspects mentioned by the farmers were an optimization of measures of pest control (19% of farmers), an improved situation of diseases (6% of farmers), and useful tips for implementation of pest control measures by farmers themselves (6% of farmers). Two out of 32 farmers assessed the approach of the project as unsuccessful due to low infestation with rodents and low reduction of the infestation.

When farmers were asked 10 to 14 months after the end of the financial contribution about the long-term collaboration of farmers with the PCOs, 24 of 33 farmers (73%) answered in the affirmative (Table 5), whereby three farmers have changed the individual PCO. Assessing the long-term collaboration of farmers with the PCOs after the end of the financial contribution in terms of frequencies of implementation of pest control measures, it appears that half of the farmers stick to the frequency specified within the project of 4–6 weeks (Table 5). This was irrespective of the fact whether the farmers stick with employing the PCO or not. In total, 20 out of 24 farmers working together with a PCO after the end of the financial contribution arranged a fixed frequency of visits ranging from “every 3-4 weeks” to “half-yearly”. Farmers that carried out pest control measures considerably more often as compared to the frequencies within the project were mostly farmers that quit working together with PCOs after financial contribution. In contrast, farmers that tend to elongate the intervals between individual pest control measures were farmers that continued working together with the PCOs (Table 5). Albeit, the Fisher exact test showed with *p* = 0.052 that the dependence of visits’ frequencies with long term collaboration with the PCO cannot be confirmed based on the chosen level of α = 0.05.

**Table 5:** Differences of the frequencies in regard to the implementation of pest control measures classified by long term collaboration categories

Long term collaboration of farmers with PCOs		
	Yes	No
Total	24 (72.7%)	9 (27.3%)
Current frequencies of implementation of control measures compared to frequencies during the project		
Higher	1 (4.2%)	3 (33.3%)
Equal	13 (54.2%)	5 (55.6%)
Lower	10 (41.7%)	1 (11.1%)

In assessing the time effort of individual aspects of pest control measures, it is noteworthy to mention that farmers who carry out pest control by themselves made on average low time specifications for the control of bait boxes (10.2 h ± 12.7 h) and the documentation per year (4.4 h ± 5.7 h). For farmers who hired a PCO, in contrast, the estimated duration for the control of bait boxes (16.2 h ± 12.6 h) and the documentation per year (9.6 h ± 9.2 h) was statistically significantly higher (one-tailed Kruskal-Wallis test). For the purchase of toxin (bait) and component parts, farmers with and without PCO generally estimated low temporal durations (1.3 h ± 1.6 h and 0.6 h ± 0.9 h, respectively). Considerably more time for training was quoted by farmers who did not engage a PCO in contrast to farmers who did, without being statistically significant. Average time specifications per year for training were 3.8 h ± 9.8 h for farmers with PCO and 20.3 h ± 44.7 h for farmers without PCO.

**Fig. 4:**
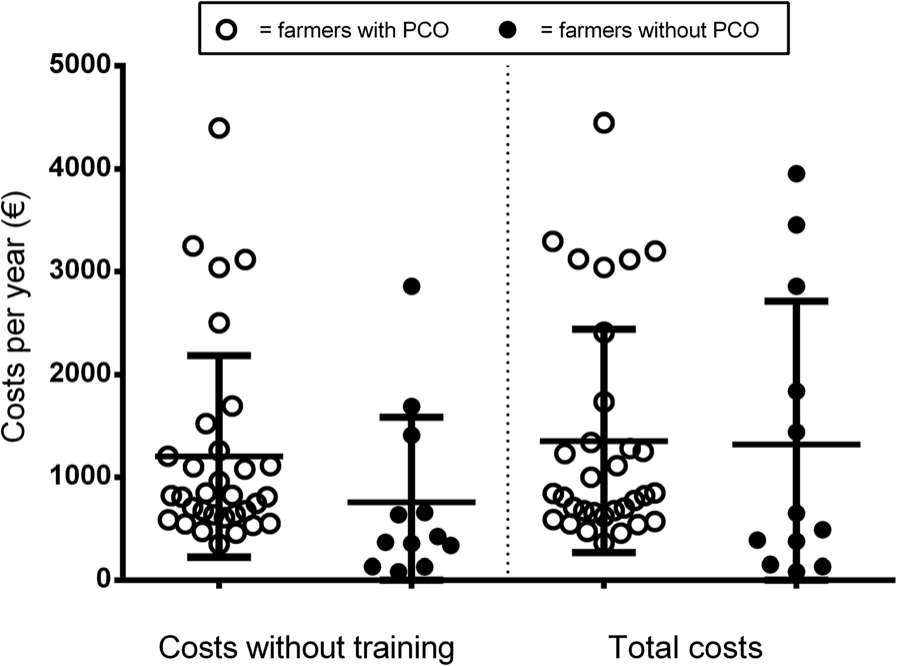
Costs of pest control measures per year estimated by farmers [farmers with PCO (○); farmers without PCO (●)]. In addition to individual values, mean ± SD is shown

Total costs of pest control measures per operation were calculated from the temporal efforts, the hourly wage rates, and the costs of materials estimated by the individual farmers. Differences in total costs occurred between the two groups (farmers with and without PCO) when time specifications for training were not considered (Fig. 4). Calculated total costs of pest control measures are on average 1.204 € per year for farmers who hired a PCO and 756 € per year for farmers who carried out pest control measures by themselves. If, however, time specifications for training had been considered, there is only a low difference in the calculated costs between farmers with (1.310 € per year) and without (1.217 € per year) PCO (Fig. 4). If one compares the calculated costs and the average cash based costs incurred by the PCOs during the project (x̅ = 1.384 € / year), the differences are minor. One year after the end of the project, the cash based costs of pest control measures dropped by 20% and amounted on average 1053 € per operation and year.

The relation between the level of participation in the project and the operational and personal characteristics of the operations as well as key figures of pest control measures are summarized in Table 6. Regarding the relation between the operational characteristics and the level of project participation, long term participating farmers tend to have higher numbers of enlarged sow breeding farms. There was the tendency of a lower proportion of piglet breeding farms and a higher proportion of fattening farms in the group of farmers not continuing collaboration with a PCO after the financial support. Regarding the number of animals per operation and the livestock units, there is the tendency that farms in the group of non-participating farms are generally slightly larger. This issue is statistically significant for piglet breeding farms (Table 6).

**Table 6:** Farm characteristics, socio-demographic details of farmers, and key figures of pest control measures classified by the level of employment of the PCO

Group	Project participation		Non-participation	
	Long-term	Only during project time	–	Total
Number	24	9	12	45
Proportion, %	53.3	20.0	26.7	100
Farm characteristics				
Sow breeding, %	20.8	11.1	16.7	17.8 ^n.s.^
Piglet breeding, %	25.0	11.1	25.0	22.2 ^n.s.^
Fattening farms, %	54.2	77.8	58.3	60.0 ^n.s.^
Total, %	100	100	100	100
No. of sows, x̅	367	350	500	385 ^n.s.^
No. of piglets, x̅	1109^a^	330^a^	1757^b^	1163*
No. of fattening pigs, x̅	1743	1885	2813	2009 ^n.s.^
Livestock units, x̅	284	318	323	301^n.s.^
Socio-demographic details on farmers				
Age (years), x̅ (med)	49 (48)	48 (49)	47 (45)	48 (47.5)^n.s.^
Number of women, %	8.3	11.1	0.0	6.7^n.s.^
Vocational training course, %	4.2	11.1	0.0	4.4^n.s.^
State certified farmer or technician, %	54.2	33.3	75.0	55.6^n.s.^
University degree, %	29.2	44.4	16.7	28.9^n.s.^
Professional training total, %	91.7	88.9	91.7	88.9^n.s.^
Key figures of pest control measures				
Infestation, x̅	2.9	3.0	–	2.9^n.s.^
No. of bait stations, x̅	24.2	24.3	17,1	21.9^n.s.^

Comment: significance level: *n.s.* non-significant; * < 0.05

Groups with different letters (a, b) differ significantly at the 5% significance level

Regarding the personal characteristics of the farmers, no significant differences in terms of age distribution between the three groups appeared. Overall, the proportion of women is low. However, the proportion of women in participating farms tends to be higher. With regard to professional training, 90% of the farmers of all groups have a professional qualification in agriculture. The proportion of farmers with vocational training courses in general is low, but tends to be higher in the group of farmers not continuing collaboration with a PCO after the financial support and is lowest for non-participating farmers. 75% of the non-participating farmers have a degree as state certified farmer or technician, whereas in the group of long-term participating farmers only 54% and in the group of farmers not continuing collaboration with a PCO after the financial support only 33% of farmers have this certification. In contrast, the proportion of farmers with a university or university of applied sciences degree is highest for farmers, who did not continue collaboration with a PCO after the financial support (Table 6).

Regarding the key figures of pest control, there were no significant differences between the three groups (Table 6).

## Discussion

Pest control measures are mandatory for pig husbandry by law in Germany (Schweinehaltungshygieneverordnung and Regulation (EC) No 852/2004 of the European Parliament and of the Council of 29 April 2004 on the hygiene of foodstuffs). In addition, for quality assurance systems like QS (Qualität und Sicherheit, GmbH) in Germany, control-routines and documentation of their implementation are required. Despite the legislative regulation and the known risk of pathogen spread to agricultural livestock by rodents, studies about the implementation practices of rodenticide pest control measures in pig farming in Germany are missing. As most farmers are professional users with qualifications, they carry out pest control measures by themselves. This is also the case in the study population of farmers where 85% of farmers controlled rodents without employing a PCO before the project. Despite these efforts results of the current study showed that half of the pig farms had problems with rodent pests. Thereby, the infestation was estimated as high by professional control operators in one third of the units. A drawback of data collection was that due to a lack of objective and easy-to-employ measurement approaches the extent of infestation was subjectively assessed by the PCOs. As PCOs were already employed at the time of estimating the extent, it is not likely, although it is possible, that PCOs tend to overestimate the problem to generate business. Bait consumption could have been ideally used as a more objective measure, but due to the focus of the study it was not directly measured. Instead, applied quantities indirectly reflect the estimation of the extent of infestation, thereby confirming roughly the estimation of the PCOs.

On more than half of the farms, no bait boxes were present at all. Based on results of the exposure assessments, rats presented the highest probability of exposure of pathogens to domestic pigs [22]. Therefore, the fact that 86% of pig farms were consistently infested with rats was alarming and calling for immediate action. Hence, it was not surprising that due to the financial support, 70% of farmers participated in the project and 53% of farmers continued engaging a PCO even after the project. Another aspect to mention is that the farms in the current study with livestock units ranging from 72 to 1040 (x̅ = 301) were comparatively large when compared with livestock units of farms from North Rhine-Westphalia. In context of the rapid structural changes taking place in livestock farming in Germany, the study farms represent growth- and future-oriented farms. Since participants were chosen on their willingness to participate, self-selection bias cannot be excluded. We assume the implementation of pest control measures is even worse on smaller, more traditional and less cooperating pig farming units. Therefore, rodent control has to be investigated further in future research.

In addition, an intensive interaction with the farmers was an important issue during the project course. Therefore, the study had to be conducted in a region accessible from South Westphalia University and thus was limited to North Rhine-Westphalia. As farms are located in one of the most intensive livestock regions in Europe, the restriction to the region of North Rhine-Westphalia doesn’t have to be necessarily a limitation. In contrast, based on the farm and farmer characteristics of the study sample, project farmers indicate to represent growth- and future-oriented farms in the European countries.

In this study, the selection of a PCO from an existing list was mainly based on the distance of the pest control operator to the location of the farm. Advantages of short distances are obvious. If necessary, the PCO is able to visit the farm in a short time and travel costs are low. Short distances are also important for PCOs. This was shown in one case after the end of the project when a PCO canceled his contract with the farmer because of long distances. Short distances are a relevant criterion for long-term collaboration for both farmers and PCOs. Furthermore, farmers took the perception of a PCO into consideration. Key values were sympathy, reliability, and a solid reputation. Background of the relevance of these values is that external parties basically pose a risk for biosecurity in pig farms. Pest control measures in the barns by PCOs are a highly sensitive issue. The project helped to overcome this impediment in cooperation between farmers and PCOs by building mutual confidence. In this context, the recommendation by colleagues is an important issue. If a PCO is working effectively on a farm, other farmers find out about the good collaboration by exchange and, as a result, prefer this particular PCO. Word-of-mouth communication within informal farmer networks therefore is central to professional rodent control in pig farming.

One major point in performing pest control measures is the correct assessment of infestation. An underestimation of the extent of an infestation is considered to be the most common reason for failure of control operations [23, 24]. Therefore, it is an important achievement that farmers learned to read early signs of infestation, like traces in the form of remnants of feeding and droppings, before the infestation is manifested by collaboration with the PCO during the project course. Because of this, pest control measures can be started considerably earlier, which ensures a better implementation of measures. In addition, removal of building defects, waste and garbage, and uncontrolled plant growth further facilitates successful implementation of pest control measures.

Besides implementing preventive measures, PCOs used anticoagulant rodenticides against pest infestations on all farms. In general, there are only few comparable values of precise quantities used against rodent pests in livestock farming in Europe. A study from Scotland shows a decrease in usage of FGARs through the years [14]. In accordance with the data of the current study, Coumatetralyl was the active ingredient used in highest quantities. The drop of application of Diphacinone and Warfarin, described from 2006, was also consistent with our data. In contrast to the data of our study, the SGARs Bromadiolon and Difenacoum were used most frequently in Scotland [14]. PCOs in the present study used most commonly Brodifacoum. The usage of Brodifacoum in Scotland is restricted to indoor areas whereas in Germany, Brodifacoum can be applied by professional users outside, around buildings and barns, too [14, 21]. The wider range of application could have led to increased use. In addition, the present study was conducted in an area in which studies had confirmed the presence of rats and mice with reduced susceptibility to one or more anticoagulant rodenticides [17, 25]. Therefore, the usage of the highly effective anticoagulant rodenticide Brodifacoum provides benefits. However, mapping the farm locations to resistance areas of the RRAC shows that Brodifacoum was frequently used in areas that are marked as areas which have no risk or are at low risk for resistance. In areas plotted as fields with medium and high levels of resistance risk, Brodifacoum was also used most commonly. In one case, on a farm with high resistance risk, Difenacoum was used most frequently. If PCOs, working in areas where resistances might occur as in North-West-Germany, administer the highest potent anticoagulant available at present to avoid failure of pest control, or if there are more unknown resistance areas present in Germany today, cannot be distinguished by the present data. In addition, a comparison of administered active compounds by farmers themselves and PCOs in pig farming is not possible as data is missing for these farmers. Unfortunately, the sample size in the present study is rather small, but with practical orientation. Therefore, applied quantities should be interpreted with caution and must be read as minimal administered values as additional baiting by the farmers cannot be excluded. Nevertheless, to our knowledge this is the first data about quantities of active ingredients of anticoagulant rodenticides used in livestock farming in Germany. In conclusion, the observed quantities of Brodifacoum used in the present study mandate further analyses of implementation practices of farmers and PCOs in livestock farming in Germany. The applied quantities indicate that more attention should be given to environmental precautions and resistance management.

Time duration of partial aspects of pest control measures as the control of bait stations and documentation per year, is estimated to be higher by farmers who hire a PCO than by farmers who carry out pest control by themselves. One possible reason behind this is that farmers cooperating with a PCO appreciate the work of the PCO and respect pest control as independent and knowledge intensive work. In contrast, farmers without PCO reported that pest control measures can be done in the course of the daily stable work which takes very little time. Time duration estimated for training was greatly scattered. Farmers without a PCO spend, according to their own statements, on average considerably more time for training; this is a necessary and desired consequence as they miss the steady, professional exchange with a PCO and cannot delegate knowledge acquisition to the PCOs. The calculation of total costs of pest control measures shows effectively how pig farmers – despite the widespread opinion that the professional would not be worth it – benefit from the work of the PCO and that there are only marginal differences between calculated total costs for employment of a PCO and cost that arise by farmers themselves. Predominantly, through the employment of a PCO farmers gain time and improved rodent control measures.

## Conclusion

Most of the farmers participating in the project employ the PCOs permanently, even beyond the end of the financial start-up contribution. Based on this study, the following practical conclusions can be provided: (1) The instrument of temporal start-up, financing professional pest control supplemented by financial self-participation and specification of professional methodology requirements, is able to ensure engagement of the PCO after the project period. This could possibly lead to long-term effects on the individual farm hygiene and on disease prevention. (2) PCOs can help to implement preventive measures on pig farms and give valuable hints for pest control. However, due to the quantities of Brodifacoum used in the present study and the resulting risk for the environment, we recommend to further analyze the implementation practices of farmers and PCOs in livestock farming in Germany. (3) Despite the widespread opinion that the professional would not be worth it – farmers financially benefit from the work of the PCO. In terms of this knowledge, we recommend to rethink existing pest control measures of farmers and to be open to collaboration with PCOs. This will finally lead to an improvement in animal health and in epizootic disease prevention.

## Additional file

Additional file 1: **Figure S1.** Distribution of project farms in twelve districts of North Rhine-Westphalia in Germany. The small picture shows whole Germany with North Rhine-Westphalia in grey. (EPS 542 kb)

## Abbreviations

AR: Anticoagulant rodenticide
FGARs: First generation anticoagulant rodenticide
n.s.: Non-significant
PCO: Pest control operator
RRAC: Rodenticide resistance action committee
SGARs: Second generation anticoagulant rodenticide
TSK: Tierseuchenkasse (Animal disease fund)
